# Phase I Trial of GD2.CART Cells Augmented With Constitutive Interleukin-7 Receptor for Treatment of High-Grade Pediatric CNS Tumors

**DOI:** 10.1200/JCO.23.02019

**Published:** 2024-05-21

**Authors:** Frank Y. Lin, Austin Stuckert, Candise Tat, Mark White, Lucia Ruggieri, Huimin Zhang, Birju Mehta, Natalia Lapteva, Zhuyong Mei, Angela Major, Sachin Thakkar, Thomas Shum, Kathan Parikh, Meng-Fen Wu, Holly B. Lindsay, Lauren Scherer, Meghan Shekar, Patricia Baxter, Tao Wang, Bambi Grilley, Karen Moeller, John Hicks, Angshumoy Roy, Jamie Anastas, Fatema Malbari, Guillermo Aldave, Murali Chintagumpala, Susan Blaney, D. Williams Parsons, Malcolm K. Brenner, Helen E. Heslop, Cliona M. Rooney, Bilal Omer

**Affiliations:** ^1^ Texas Children's Cancer Center, Baylor College of Medicine, Houston, TX; ^2^ Dan L Duncan Comprehensive Cancer Center, Houston, TX; ^3^ Department of Neurosurgery, Baylor College of Medicine, Houston, TX; ^4^ Center for Cell and Gene Therapy, Baylor College of Medicine, Texas Children's Hospital and Houston Methodist Hospital, Houston, TX; ^5^ Department of Pathology, Baylor College of Medicine, Houston, TX; ^6^ Department of Radiology, Brigham and Women's Hospital, Boston, MA; ^7^ Department of Medicine, Baylor College of Medicine, Houston, TX; ^8^ Department of Pediatrics Heme-Onc and Bone Marrow Transplantation, Children's Hospital Colorado Center for Cancer and Blood Disorders, University of Colorado Anschutz Medical Campus, Denver, CO; ^9^ Department of Radiology, Baylor College of Medicine, Houston, TX; ^10^ Department of Neurology, Baylor College of Medicine, Houston, TX

## Abstract

**PURPOSE:**

T cells modified with chimeric antigen receptors (CARTs) have demonstrated efficacy for hematologic malignancies; however, benefit for patients with CNS tumors has been limited. To enhance T cell activity against GD2+ CNS malignancies, we modified GD2-directed CART cells (GD2.CARTs) with a constitutively active interleukin (IL)-7 receptor (C7R-GD2.CARTs).

**METHODS:**

Patients age 1-21 years with H3K27-altered diffuse midline glioma (DMG) or other recurrent GD2-expressing CNS tumors were eligible for this phase I trial (ClinicalTrials.gov identifier: NCT04099797). All subjects received standard-of-care adjuvant radiation therapy or chemotherapy before study enrollment. The first treatment cohort received GD2.CARTs alone (1 × 10^7^ cells/m^2^), and subsequent cohorts received C7R-GD2.CARTs at two dose levels (1 × 10^7^ cells/m^2^; 3 × 10^7^ cells/m^2^). Standard lymphodepletion with cyclophosphamide and fludarabine was included at all dose levels.

**RESULTS:**

Eleven patients (age 4-18 years) received therapy without dose-limiting toxicity. The GD2.CART cohort did not experience toxicity, but had disease progression after brief improvement of residual neurologic deficits (≤3 weeks). The C7R-GD2.CART cohort developed grade 1 tumor inflammation–associated neurotoxicity in seven of eight (88%) cases, controllable with anakinra. Cytokine release syndrome was observed in six of eight (75%, grade 1 in all but one patient) and associated with increased circulating IL-6 and IP-10 (*P* < .05). Patients receiving C7R-GD2.CARTs experienced temporary improvement from baseline neurologic deficits (range, 2 to >12 months), and seven of eight (88%) remained eligible for additional treatment cycles (range 2-4 cycles). Partial responses by iRANO criteria were observed in two of seven (29%) patients with DMG treated by C7R-GD2.CARTs.

**CONCLUSION:**

Intravenous GD2.CARTs with and without C7R were well tolerated. Patients treated with C7R-GD2.CARTs exhibited transient improvement of neurologic deficits and increased circulating cytokines/chemokines. Treatment with C7R-GD2.CARTs represents a novel approach warranting further investigation for children with these incurable CNS cancers.

## INTRODUCTION

Novel therapeutic approaches are needed for children with diffuse midline glioma (DMG) and other treatment-refractory CNS tumors, who experience a dismal prognosis with conventional treatment modalities.^1-3^ Adoptive cellular immunotherapy using T cells modified with chimeric antigen receptors (CARTs) is one such strategy.^4-6^ However, CART efficacy for CNS tumors has been constrained by an immunosuppressive tumor microenvironment (TME) characterized in part by limited availability of immunostimulatory cytokines.^7-11^ To overcome the hostile tumor milieu, we previously identified a constitutively active interleukin (IL)-7 receptor (C7R) variant that activates downstream STAT5 pathway signaling independent of IL-7, without affecting bystander immune cells.^12^ C7R can thereby enhance CART survival, proliferation, and function in the absence of cytokine during repeated antigen exposure in vitro and in murine models.

**CONTEXT**

**Key Objective**
Can a constitutively active interleukin-7 receptor (C7R) safely augment antitumor activity of chimeric antigen receptor (CART) cells targeted against the antigen GD2 (GD2.CART) for children with high-grade GD2-expressing CNS tumors?
**Knowledge Generated**
Among patients treated with C7R-GD2.CART cells, seven of 8 (88%) developed grade 1 tumor inflammation–associated neurotoxicity (TIAN) and six of eight (75%) cytokine release syndrome (grade 1 n = 5, grade 4 n = 1), controlled with anticytokine agents in all patients. Seven of these eight (88%) patients received multiple cycles and exhibited higher levels of granzyme B and interferon-γ expression in peripheral blood compared with patients treated with GD2.CART cells without C7R.
**Relevance *(S. Bhatia)***
This study shows evidence that optimized CARTs are safe in children with bad prognosis CNS tumors and this novel approach warrants further investigation for these children.*
  *Relevance section written by *JCO* Associate Editor Smita Bhatia, MD, MPH, FASCO.

The disialoganglioside GD2 is highly expressed in pediatric DMG harboring histone H3.3 or H3.1 complex aberrations (H3K27-altered)^13^ and some recurrent embryonal CNS tumors, providing a target for CART cells (GD2.CARTs). GD2.CARTs had encouraging activity against GD2-expressing tumors in both preclinical studies and early-phase clinical trials for children with neuroblastoma.^14-17^ More recently, GD2.CARTs have been evaluated in four patients with CNS tumors, with improvement observed in three.^18^ We conducted a phase I trial to examine whether C7R can safely augment the antitumor activity and function of GD2.CARTs. We evaluated the safety and efficacy of intravenously administered GD2.CARTs cotransduced with C7R (C7R-GD2.CARTs) for treating H3K27-altered DMG and other recurrent GD2-expressing pediatric CNS tumors. We present our trial design, safety data, and clinical and tumor imaging response findings, offering insights into the feasibility and therapeutic potential of combining a GD2.CAR with C7R in T cells for the treatment of children with DMG and other GD2-expressing CNS tumors.

## METHODS

### Study Design and Eligibility

The trial was approved by the institutional biosafety committee and institutional review board of Baylor College of Medicine, and the US Food and Drug Administration (ClinicalTrials.gov identifier:NCT04099797). Written informed consent (and assent when applicable) was obtained for all patients in two stages, first for procurement of patient tissues and subsequently for protocol therapy.

Patients age 12 months to 21 years with newly diagnosed H3K27-altered DMG (pontine or thalamic), recurrent H3K27-altered DMG (except pontine), or other recurrent/progressive GD2-expressing CNS tumors were eligible for study after molecular confirmation of a tumor H3K27 alteration from a Clinical Laboratory Improvement Amendments– and College of American Pathologists-certified laboratory (for DMG) or immunohistochemical demonstration of GD2 expression (in >50% of tumor cells) at Texas Children’s Hospital (TCH). All patients were required to complete standard-of-care radiation therapy at least 4 weeks before CART therapy. Additional eligibility criteria included tumor size ≤5 cm in greatest dimension (or ≤5.5 cm for debulked tumors) on preconsent magnetic resonance imaging (MRI), Karnofsky or Lansky performance score ≥50, and stable neurologic examination on a stable steroid dose for ≥7 days. A ventricular catheter (Ommaya reservoir or ventriculoperitoneal shunt) was required as a safety precaution for all patients before CART therapy.

**FIG 1.**
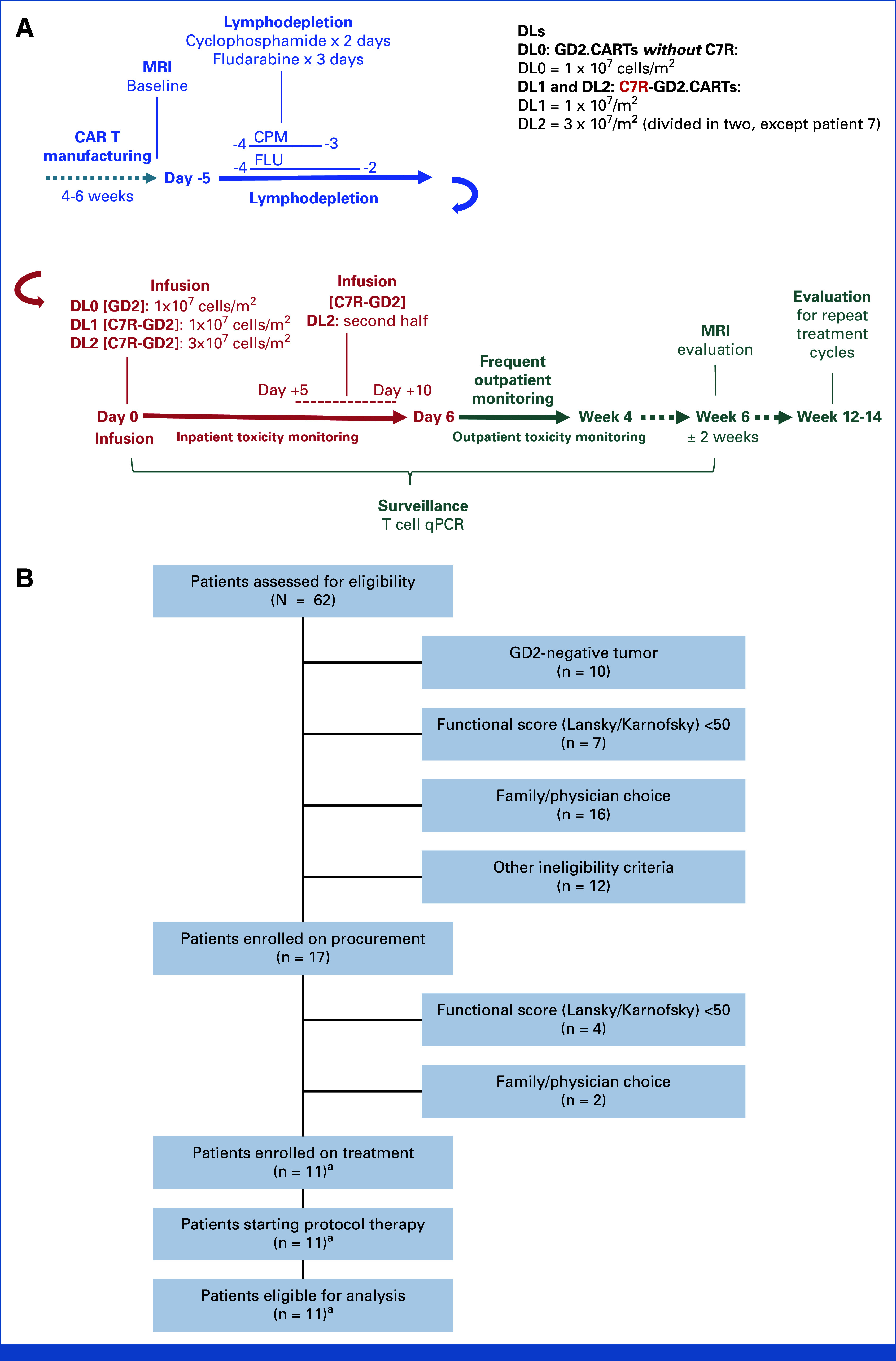
Study design and patient screening. (A) Study schema. (B) Patient flow diagram of GAIL-B. Other reasons for ineligibility included tumor size and progressive pontine DMG. ^a^One patient with DMG demonstrating partial response on dose level 1 re-enrolled onto dose level 2. CART, chimeric antigen receptor; DL, dose level; DMG, diffuse midline glioma H3K27-altered; MRI, magnetic resonance imaging; qPCR, quantitative polymerase chain reaction.

The trial used a standard 3 + 3 design. All subjects received standard lymphodepletion with fludarabine (30 mg/m^2^ once daily for three doses) and cyclophosphamide (500 mg/m^2^ once daily for two doses). The first treatment cohort (dose level 0, DL0) received intravenous infusion of GD2.CARTs without the C7R gene, at 1 × 10^7^ cells/m^2^. Two subsequent cohorts received C7R-GD2.CARTs: dose level 1 (DL1) kept the same cell dose (1 × 10^7^ cells/m^2^), followed by dose level 2 (DL2), which escalated to 3 × 10^7^ cells/m^2^. Patients with stable or improved disease by imaging were eligible to receive up to three additional cycles of GD2.CARTs at an identical dose at a minimum of 6-week intervals (Fig 1A). Patients treated between June 2021 and March 2023, and data collected up to May 1, 2023, are reported in this manuscript.

### Adverse Event Reporting and Toxicity Management

Dose-limiting toxicity (DLT) was specified for prolonged cytopenias and nonhematologic toxicities. All grade 4 neurotoxicity were considered a DLT, as was grade 3 or 4 cytokine release syndrome (CRS) that failed to improve to grade 2 CRS within 72 hours. Early signs of non–dose-limiting CRS and CART-associated pseudoprogression were managed with anticytokine agents (tocilizumab and anakinra), and corticosteroids were added for higher-grade toxicity, according to study protocol. Adverse events (AEs) and toxicity were reported according to the NCI Common Terminology Criteria for Adverse Events (CTCAE) version 5.0. CRS and immune effector cell-associated neurotoxicity syndrome were graded according to American Society for Transplantation and Cellular Therapy criteria.^19^ A grading system for tumor inflammation–associated neurotoxicity (TIAN)^20^ was published after the data collection period for the current trial and scoring was retroactively assigned.

### Measurement of Response

Treated patients were evaluated with longitudinal neurologic examinations by the study team (daily while hospitalized, then 3 times weekly for the first 2 weeks after infusion, twice during the third week, and once during the fourth week) and by MRI examination at 6 weeks and 3 months after CART infusion. Tumor disease evaluation was obtained before each subsequent CART treatment cycle. Objective tumor response, including best overall response, or progression was determined using the iRANO^21^ criteria for CNS tumors to account for the possibility of immunotherapy-associated pseudoprogression. Transient neurologic changes consistent with CART-associated pseudoprogression/TIAN were not considered for response assessment. Clinical response was assessed by patient/parent report and physical examination during the longitudinal study examinations, and defined as improvement of at least one preexisting neurologic deficit (measured by a decrease of at least one grade according to CTCAE version 5) without progression of other baseline symptoms, or development of new neurologic deficits. Clinical response was deemed to have ceased if the relevant condition reverted to baseline or worse.

### Statistical Considerations

Dose escalation followed a standard 3 + 3 design with a 4-week toxicity period after infusion. Duration of clinical response was defined as the time from initial clinical improvement after infusion to the date of radiographic or clinical progression.

### CART Manufacturing, Administration, and Monitoring

We collected autologous peripheral blood mononuclear cells (PBMCs) from 60 mL of peripheral blood to manufacture GD2.CART and C7R-GD2.CART cell lines in the Cell and Gene Therapy Good Manufacturing Practice facility (Houston, TX). To generate CARTs, isolated PBMCs were activated with clinical-grade CD3 and CD28 monoclonal antibodies, sequentially cotransduced with the GD2.CAR and C7R retroviral vectors, and expanded in the presence of IL-7 and IL-15.^12^ Cell aliquots were removed for standard release testing and the remaining cells cryopreserved. For patient administration, autologous CARTs were thawed and infused via central venous catheter. CART persistence and expansion, and C7R detection were assessed by using real-time polymerase chain reaction (PCR),^22^ and C7R and GD2.CAR flow cytometry^12^ assays as previously described. Flow cytometry including subset analysis was performed on a BD FACSCanto 2 system (Franklin Lakes, NJ). Cytotoxicity was determined with a standard 4-hour ^51^Cr-release assay with GD2-expressing tumor cell targets.^23^

### GD2 Immunohistochemistry and Immunofluorescence

Tumor tissues were assessed for GD2 expression by immunohistochemical staining performed on a BOND-III stainer (Leica Biosystems, Deer Park, IL) and using a BOND Polymer Refine Red Detection system (Leica Biosystems, Deer Park, IL). A 3F8 antibody (provided by Dr Nai-Kong Cheung, MSKCC, New York City, NY) was used at 1:2,000 dilution and tumors were considered GD2-positive if >50% of cells demonstrated positive staining. Immunohistochemical staining and scoring was performed by the study pathology technician (A.M.) and pathologist (J.H.) for all patients with available tissue.

### Tumor Molecular Profiling

When available, tumor DNA was subjected to methylation profiling using an Illumina DNA Methylation EPIC array (San Diego, CA) and classified using a random forest prediction model assembled from a published pediatric CNS tumor reference as previously described.^24,25^

### Multiplex Cytokine Immunoassay

A human cytokine magnetic 46-plex panel kit assay was performed on the Luminex 100/200 (Luminex xMAP Technology; Luminex, Austin, TX) following the manufacturer's protocol (R&D Systems, Minneapolis, MN) to quantify analytes in patient plasma samples. Plasma was obtained from patients before initial CART infusion and at 3 hours, 1 week, 2 weeks, and 4 weeks after infusion, then maintained at -80°C until the assay was performed.

## RESULTS

### Patient Characteristics

Sixty-two patients were screened and 33 met eligibility criteria for procurement (Fig 1B). Among these 33 patients, 17 enrolled and provided blood samples for generation of CART cell lines. Eleven of these 17 patients proceeded to receive CART therapy; of the other six patients, four experienced clinical deterioration before treatment, and two elected other experimental trials or no further therapy (Fig 1B). All treated patients were evaluable for response and toxicity assessment. One patient with thalamic DMG re-enrolled at DL2 (C7R-GD2.CART, 3 × 10^7^ cells/m^2^) of the trial after tolerating two treatment cycles on DL1 (C7R-GD2.CART, 1 × 10^7^ cells/m^2^).

The demographics of treated subjects are shown in Table 1. The median age of patients treated was 12 years with a near-even ratio of female to male patients. Ten of 11 (91%) patients had neurologic deficits at enrollment (Appendix Table A1, online only). The most prevalent diagnosis was DMG (n = 8, thalamic: 4, pontine: 3, spine: 1), followed by recurrent medulloblastoma (n = 2) and recurrent atypical teratoid/rhabdoid tumor (AT/RT; n = 1). Three of four patients with thalamic DMG had recurrent disease (patients 3, 9, and 12). All patients received radiation therapy before CARTs (median time from radiotherapy to CART infusion was 10 weeks for DMG and 38 weeks for embryonal tumors, Table 2). Two patients with recurrent thalamic DMG had received radiation therapy and chemotherapy before enrollment on study, while all other patients with DMG received CART infusions within 3 months of radiation therapy. Both patients with recurrent medulloblastoma had undergone multiple regimens of chemotherapy, and the patient with recurrent AT/RT received upfront adjuvant chemotherapy and craniospinal reirradiation therapy before CART infusion (Appendix Table A1).

**TABLE 1.** Patient Demographics

Characteristic	DL0 (n = 3)	DL1 (n = 3)	DL2 (n = 6^a^)	Total (N = 11)
Age, years, median (range)	13 (4-17)	11 (11-14)	12 (10-18)	12 (4-18)
Sex, No.				
Female	1	2	3	5^a^
Male	2	1	3	6
Ethnicity, No.				
Non-Hispanic	3	3	2	7^a^
Hispanic	0	0	4	4
Race, No.				
White	1	2	3	6
Black or African American	0	0	1	1
Asian	2	0	0	2
More than one race	0	1	1	1^a^
Not reported/unknown	0	0	1	1
No. of infusions, median (range)	1	2 (2-4)	2 (1-3)	2 (1-4)
Diagnosis, No.				
DMG				
Thalamic	1	1	3	4^a^
Pons	0	1	2	3
Spine	1	0	0	1
Other				
MB	0	1	1	2
ATRT	1	0	0	1

Abbreviations: ATRT, atypical teratoid rhabdoid tumor; DL, dose level; DMG, diffuse midline glioma; MB, medulloblastoma.

^a^ One patient with DMG initially treated with two treatment cycles on DL 1 was re-enrolled onto DL 2.

**TABLE 2.** Clinical Outcomes

Patient No.	Dose Level	Diagnosis	Location	Upfront or Progressive	GD2 IHC	Product	Dose, Cells/m^2^	Days From XRT^a^	CRS Grade	TIAN Grade	Clinical Response	Duration of Clinical Response	Radiographic Response (at 6 weeks)	Best Radiographic Response	No. of Infusion Cycles
1	0	DMG K27-altered	Thalamic	Upfront	Pos	GD2.CAR	10 million	90	0	0	Improved lower-extremity strength	3 weeks	PD	PD	1
2	0	ATRT	Spine	Progressive	Pos	GD2.CAR	10 million	268	0	0	Mildly improved upper-extremity strength	2 weeks	PD	PD	1
3	0	DMG K27-altered	Spine	Progressive	NA	GD2.CAR	10 million	61	0	0	Improved gait stability	2-3 weeks	PD	PD	1
4	1	Medulloblastoma	PF, Spine	Progressive	Pos	C7R-GD2.CAR	10 million	208	1	1	Resolved incontinence, improved strength	2 months	SD	SD	2
5/10^b^	1/2	DMG K27-altered	Thalamic/tectal	Upfront	NA	C7R-GD2.CAR	10 million/30 million^c^	58/310	0	1	Improved diplopia, stable gait, weaned off steroids, decreased headache	27 months^b^	SD	PR	2 + 2^b^
6	1	DMG K27-altered	Pons	Upfront	NA	C7R-GD2.CAR	10 million	71	1	1	Resolved facial nerve palsy, regained stair climbing ability	6 months	SD	SD	4
7	2	Medulloblastoma	Spine	Progressive	Pos	C7R-GD2.CAR	30 million	1,839	4	1	Improved right dysdiadochokinesis, mild ataxia	9 months	SD	SD	3
8	2	DMG K27-altered	Pons	Upfront	Pos	C7R-GD2.CAR	30 million^c^	48	1	1	Improved diplopia, right CN7 palsy, slurred speech, dysphagia, right hemiparesis, and gait instability	5 months	PR	PR	3
9	2	DMG K27-altered	Thalamic	Progressive	NA	C7R-GD2.CAR	30 million^c^	308	1	1	Asymptomatic	4 months	SD	SD	2
11	2	DMG K27-altered	Pons	Upfront	Pos	C7R-GD2.CAR	30 million^c^	84	0	0	Improved pace of speech and gait stability	4 months	SD	SD	2
12	2	DMG K27-altered	Thalamic	Progressive	Neg	C7R-GD2.CAR	30 million^c^	62	1	1	No clinical response	0 months	PD	PD	1

Abbreviations: ATRT, atypical teratoid/rhabdoid tumor; CART, chimeric antigen receptor; CRS, cytokine release syndrome; DL, dose level; DMG K27, diffuse midline glioma H3K27-altered; MRI, magnetic resonance imaging; NA, tissue not available for testing; PD, progressive disease; PF, posterior fossa; PR, partial response; SD, stable disease; TIAN, tumor inflammation–associated neurotoxicity.

^a^ Denotes number of days between last radiation therapy to first CART infusion.

^b^ Patients 5 and 10 represent the same individual who received two infusions at DL 1, then re-enrolled to DL 2 to receive two additional infusions to date with PR of tumor. MRI appearance of tumor met definition of PR (by iRANO criteria) after the first cycle on DL2 (third overall cycle).

^c^ 30 million cells/m^2^ dose administered as split halves 5-7 days apart.

### Toxicities and Management of AEs

**FIG 2.**
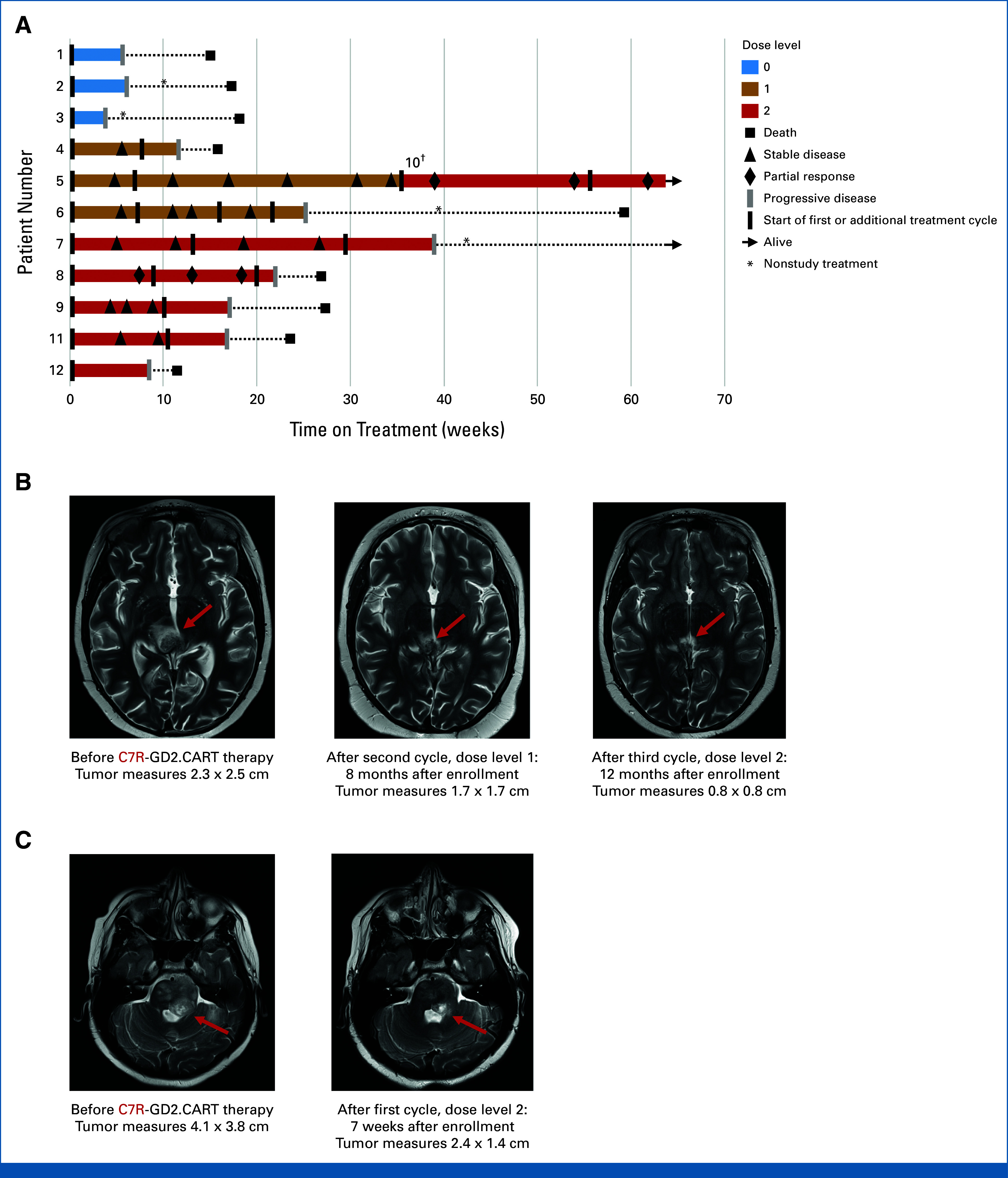
Summary of patient outcomes. (A) Swimmer plot, (B) MRI case examples (patient 5/10), and (C) MRI case examples (patient 8). Patients 5/10 and 8 vignettes are described in Appendix Table A2. MRI, magnetic resonance imaging.

**FIG A1.**
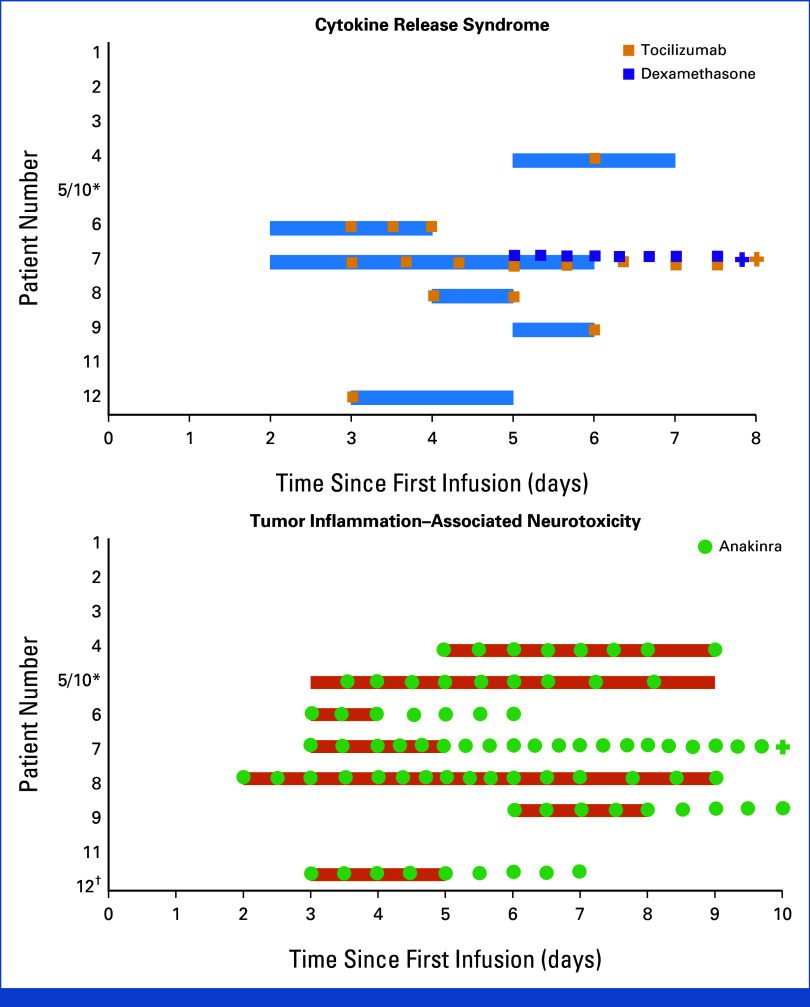
Timeline and management of (A) CRS (blue bar) and (B) TIAN (orange bar) experienced by patients during first treatment cycle. ^+^Indicates continuation of therapy. Patient 7 continued on tocilizumab through day 9, dexamethasone through day 11, and anakinra through day 12. *Patient 5/10 represents the same individual who received two infusions at DL 1, then re-enrolled to DL 2 to receive two additional infusions to date. ^†^Patient 12 exhibited neurologic symptoms likely related to tumor progression, however, attributed as grade 1 TIAN. Retrospectively tested tumor tissue was GD2-negative. CRS, cytokine release syndrome; TIAN, tumor inflammation–associated neurotoxicity.

The 11 patients received a total of 24 CART infusions. Seven of the eight (88%) patients treated with C7R-GD2.CARTs received multiple cycles. No DLTs were observed (Table 2, Fig 2A). None of the three patients who received GD2.CARTs without C7R (dose level 0, 1 × 10^7^ cells/m^2^) experienced CRS or TIAN. In the cohorts treated with C7R-GD2.CARTs, seven of 8 (88%) patients experienced grade 1 TIAN and five of eight (63%) grade 1 CRS (Appendix Fig A1). All instances of grade 1 CRS resolved within 24 hours after tocilizumab, and TIAN within 7 days with anakinra therapy. Intracranial pressure measured in two patients experiencing grade 1 TIAN was within normal limits. The first patient treated at DL2 (patient 7) developed grade 4 CRS 48 hours after infusion of 3 × 10^7^ cells/m^2^ C7R-GD2.CARTs, improving to grade 2 by 72 hours and resolving by the fifth day with anakinra, tocilizumab, and dexamethasone treatment. Notably, patient 7 had been heavily treated with multiple lines of chemotherapy as part of his previous treatment for medulloblastoma. As a safety precaution, all subsequent patients on DL2 received C7R-GD2.CART infusions in 1.5 × 10^7^ cells/m^2^ split doses administered 5-7 days apart. No further incidences of grade 2 or higher CRS occurred in five subsequent patients treated at DL2.

### Evaluation of Activity and Efficacy

**FIG A2.**
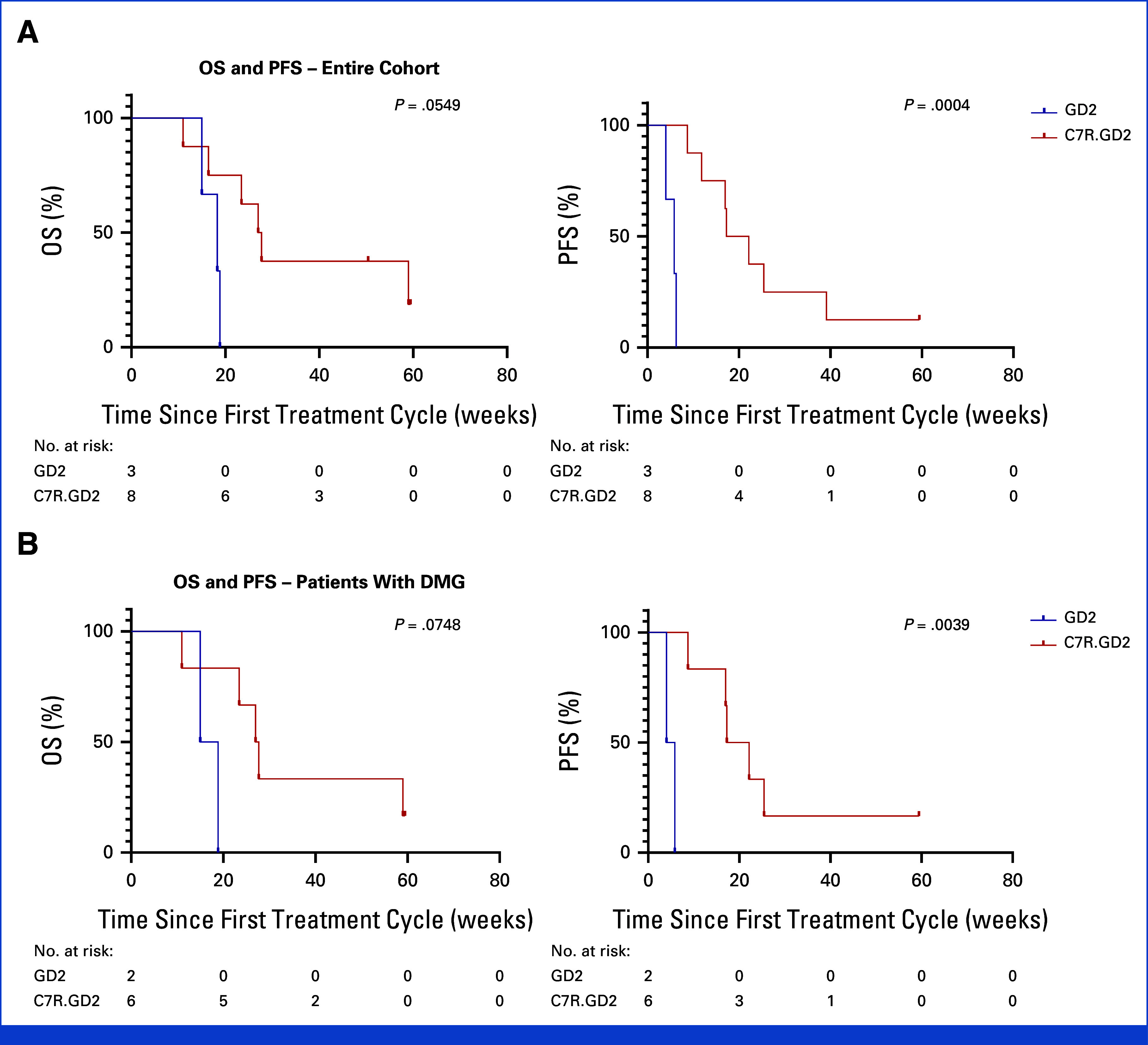
Kaplan-Meier curves demonstrating OS and PFS from time of first treatment for (A) the entire cohort and (B) patients with DMG. *P values calculated using Mantel-Cox log-rank test. DMG, diffuse midline glioma; OS, overall survival; PFS, progression-free survival.

Among the 10 patients with preexisting neurologic deficits before CART infusion, all but one (90%) experienced improvement of baseline neurologic deficits within 3 weeks of CART infusion. The duration was <3 weeks for patients receiving GD2.CARTs without C7R (DL0), but of the seven who received C7R-GD2.CARTs, 6 (86%) exhibited reduced neurologic deficits for a median of 5 months (range, 2-13 months). MRI at 6 weeks demonstrated partial response (PR) or stable disease (SD) in seven of the eight patients receiving C7R-GD2.CARTs (88%; PR, 1; SD, 6). Two of the five patients with DMG (treated upfront for thalamic and pontine DMG respectively) demonstrated PR as the best radiographic response by iRANO criteria (Figs 2B and 2C, Table 2, Appendix Table A2) and development of intratumoral necrosis was observed in three of five (60%) patients with DMG with SD. Although radiation therapy–induced pseudoprogression cannot be definitively excluded, the study baseline MRI either demonstrated smaller tumor size compared with preradiotherapy imaging, or were obtained at an extended period from the last radiation dose (Table 2). Seven of eight (88%) patients treated with C7R-GD2.CARTs (on either dose level) received additional treatment cycles (median 2.5; Table 2), while none of the patients treated with GD2.CARTs qualified for a second cycle. Progression-free survival was longer in patients treated with C7R-GD2.CARTs compared with GD2.CARTs (*P* < .005, Appendix Fig A2); however, most patients progressed eventually despite repeat CART infusions. Notably, the only patient to experience neither clinical or radiographic response (patient 12) was retroactively found to have a GD2-negative DMG after infusion.

### Cell Line Characteristics

**FIG A3.**
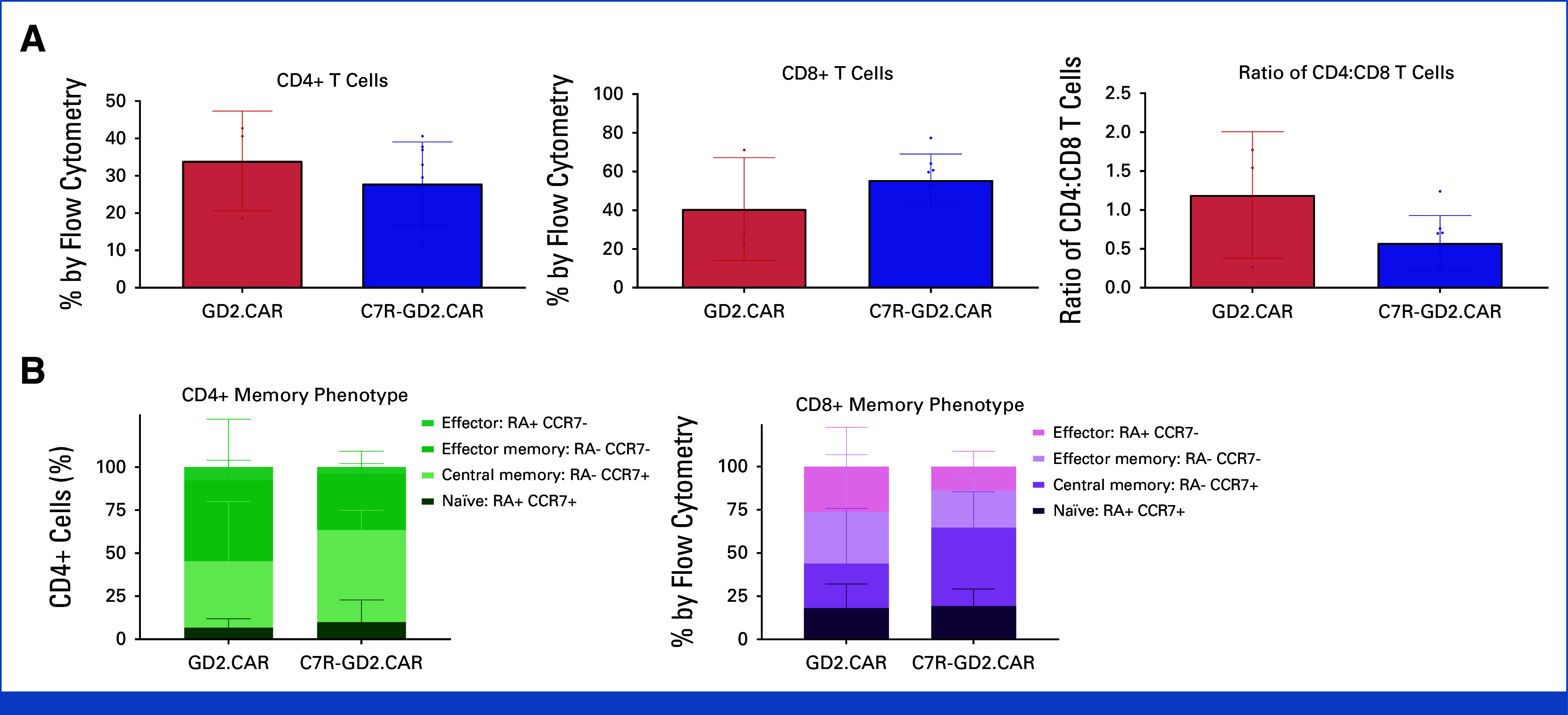
Subset analysis of infused lines. Manufactured cell lines were analyzed by flow cytometry. Dead cells were excluded by 7-AAD, and fraction of CD3^+^ cells is shown. (A) CD4 and CD8 subsets. (B) Memory phenotype.

Cell line manufacturing was successful for all 17 patients who underwent cell procurement. Several subjects had both single-transduced GD2.CAR and double-transduced C7R-GD2.CART lines manufactured. In the infused lines, mean GD2.CAR transduction was 82.0% and average double-transduction was 50.3% for C7R-GD2.CART lines (Appendix Table A3). There was a trend toward a higher CD8 to CD4 ratio and a higher proportion of central memory cells in C7R-GD2.CARTs compared with GD2.CARTs (Appendix Fig A3).

### In Vivo Detection and Persistence of C7R and GD2.CAR

**FIG 3.**
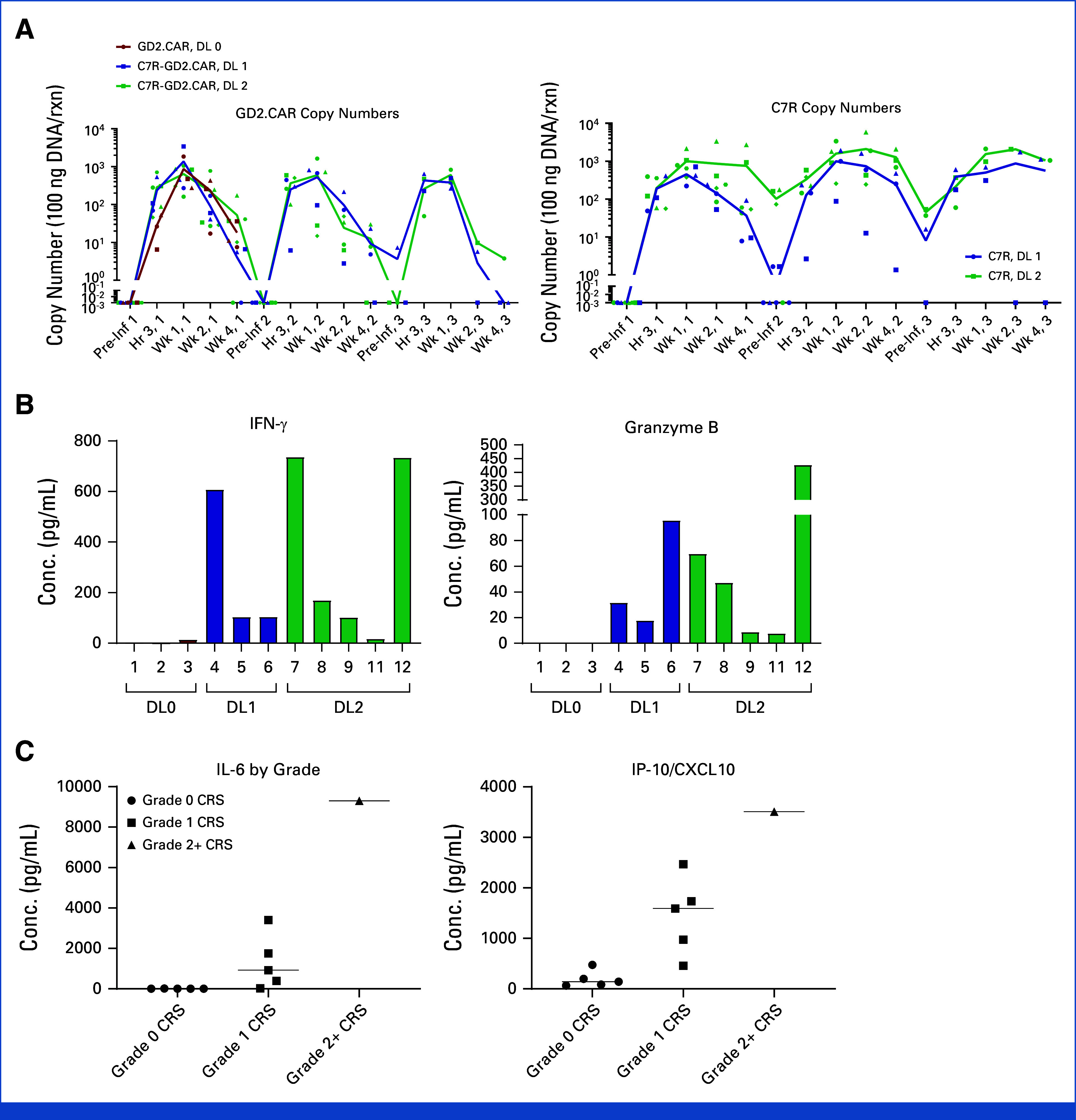
GD2 expression, CART kinetics, and cytokine production. (A) GD2.CAR and C7R cell copy numbers as measured by qPCR pre-infusion, stratified by dose level, obtained at hour 3, week 1, week 2, and week 4 for each treatment cycle. (B) Cytokine production by dose level: IFN-γ and granzyme B. (C) CRS grade and cytokine levels: IL-6 and IP-10/CXCL10. CART, chimeric antigen receptor; CRS, cytokine release syndrome; DL, dose level; IFN, interferon; qPCR, quantitative polymerase chain reaction.

**FIG A4.**
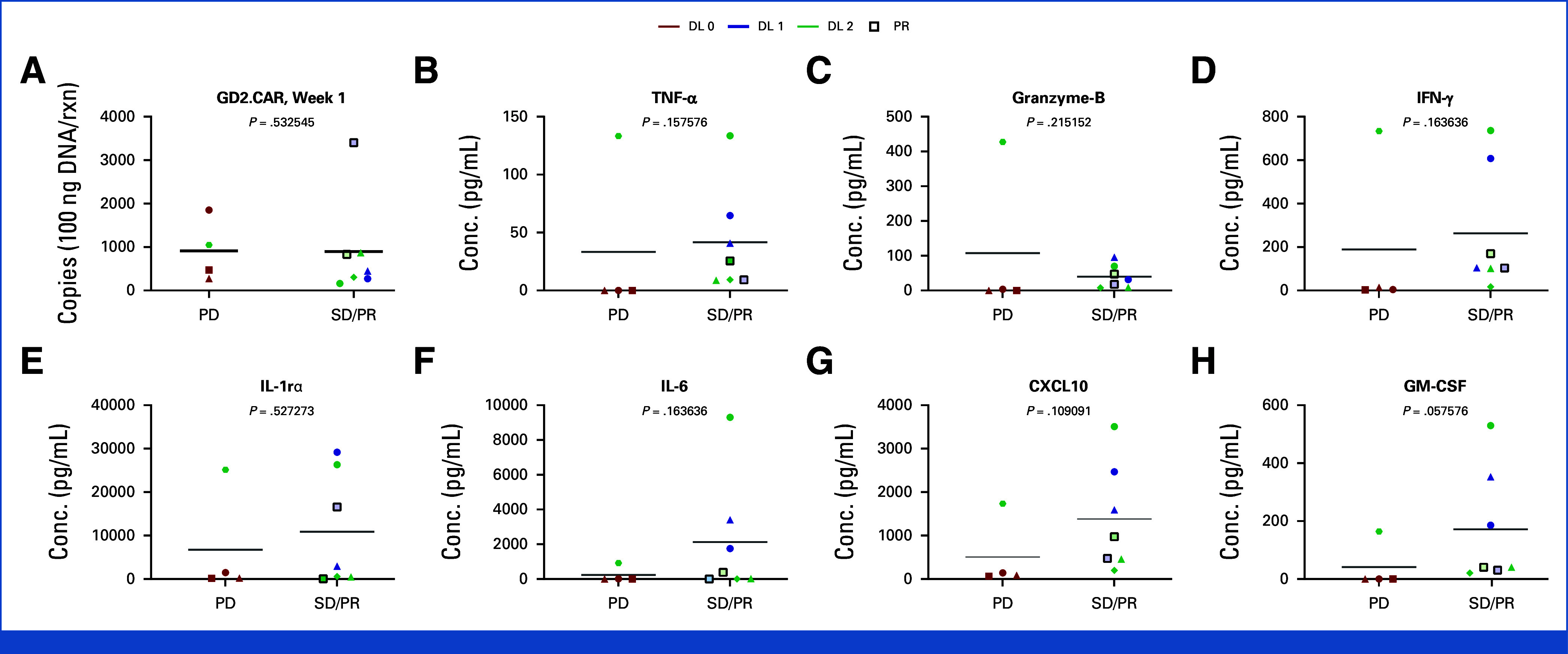
Correlation of response with (A) CART kinetics and cytokine/chemokine levels of (B) TNF-α, (C) granzyme-B, (D) IFN-γ, (E) IL-1rα, (F) IL-6, (G) CXCL10, and (H) GM-CSF. *P* values calculated using Mann-Whitney U test. CART, chimeric antigen receptor; DL, dose level; GM-CSF, granulocyte-macrophage colony stimulating factor; IFN, interferon; PD, progressive disease; PR, partial response; SD, stable disease; TNF, tumor necrosis factor.

We observed robust expansion of GD2.CAR and C7R-GD2.CARTs in patient peripheral blood as measured by quantitative PCR (qPCR) for the GD2.CAR and C7R transgenes at all dose levels with no significant relationship between peak expansion and clinical response (Appendix Fig A4A). Transgene levels peaked at week 1 with subsequent decrease (Fig 3A) and more gradual decline of C7R transgene compared with the GD2.CAR transgene. Expansion kinetics of both transgenes after second and third infusions were comparable with the first. CSF sampling was optional and was limited to a few patients. However, both the GD2.CAR and the C7R transgene were detectable in CSF collected at week 1 (patient 8) and week 2 (patient 12). Postmortem tumor was examined in one case (patient 8) 3 months after C7R-GD2.CART infusion, at which time the C7R transgene, but not GD2.CAR, was detectable by transgene analysis and flow cytometry.

### Postinfusion Cytokines and Chemokines

Cytokine profiles analyzed from pre- and post-infusion blood samples identified an elevated expression of granzyme B as well as interferon (IFN)-γ, among other cytokines, in patients treated with C7R-GD2.CARTs compared with those receiving GD2.CARTs (Fig 3B, Appendix Figs A4B-A4H). Patients who developed CRS had correspondingly higher levels of IL-6 and the chemokine IP-10 when measured at week one after infusion (*P* < .05, Fig 3C), with the single patient experiencing grade 4 CRS (patient 7) demonstrating the highest levels of both.

## DISCUSSION

This phase I study examined the safety and efficacy of C7R-GD2.CART cell therapy in children with H3K27-altered DMG and other GD2-expressing recurrent CNS tumors, diagnoses currently considered incurable. By using C7R, our study aimed to augment the expansion and functionality of CART cells in a hostile TME.

The toxicity profile of C7R-GD2.CARTs was tolerable overall, with low-grade CRS and TIAN upon C7R inclusion. Our safety procedures predated recently proposed definitions for TIAN^20^ and had a lower threshold to initiate anakinra. In our cohort, early use of intravenous anakinra effectively mitigated TIAN without the need for additional steroids, which might negatively affect the infused T cells. A single instance of grade 4 CRS occurred in the first patient on the highest dose level after the administration of C7R-GD2.CARTs as a single infusion. Implementing a fractionated dosing strategy in subsequent patients reduced toxicity, resulting only in grade 1 CRS.^26^

In patients with baseline neurologic deficits, TIAN manifested as an exacerbation of these symptoms lasting 1-7 days (Appendix Fig A1B). After the initial period of TIAN, however, the majority of patients experienced temporary improvement of the same deficits. Progression-free survival was estimated to be longer among patients treated with C7R-GD2.CARTs compared with GD2.CARTs (*P* < .005, Appendix Fig A2), and MRI revealed PR and intratumoral necrosis in several patients. However, further investigation into clinical efficacy is necessary, as this study enrolled patients with heterogeneous diagnoses and pre-enrollment treatments, tumor response was confounded by the possibility of radiotherapy-associated pseudoprogression, and was not powered to measure survival outcomes. Furthermore, all but one patient (patient 5/10, Appendix Table A2) developed eventual tumor progression despite repeated cell infusion cycles. Postmortem examination of tumor tissue from one patient with DMG demonstrated significant tumor necrosis and presence of GD2 antigen, suggesting that factors other than antigen escape may contribute to waning responses.^17,18,22^ Of note, the sole patient without clinical improvement had a DMG retrospectively discovered to be GD2-negative, despite harboring a confirmed H3K27 alteration, highlighting the need for precise patient selection on the basis of tumor antigen expression when possible.

With systemic C7R-GD2.CART administration, the GD2.CAR and the C7R transgenes were detectable in peripheral blood at initial and follow-up infusions and in CSF in limited samples. CSF sampling was optional and therefore limited in this phase of the trial, restricting assessment of expansion kinetics and cytokine levels in the CNS, which may be higher than in peripheral blood. The degree or duration of CARs detected in peripheral blood did not differ significantly with or without C7R, likely related to lymphodepletion providing sufficient cytokine for GD2.CART expansion outside the TME. However, patients treated with C7R-GD2.CARTs exhibited a higher proportion of tumor-specific polyfunctional cells as well as circulating IFN-γ and granzyme B levels, which have been associated with improved tumor killing by T cells.^27^

It is notable that early indications of clinical responses were observed in this cohort of children with extremely poor-prognosis CNS tumors, including the first pediatric patients with thalamic DMG and recurrent embryonal tumors to be treated with GD2.CARTs. Only radiation therapy has proven utility for children with DMG, who have an expected survival of little more than a year from diagnosis, and^28,29^ the prognosis of recurrent embryonal CNS tumors is similarly dismal.^3,30^ Development of immunotherapies against these CNS tumors presents particular challenges, including the blood-brain barrier, tumor heterogeneity, and a suppressive TME. Our results add to a small number of recent studies that have provided clues into potentially effective strategies,^18,31-34^ supporting further investigation towards optimizing CART effectiveness—potentially through the addition of locoregional (intraventricular) delivery and development of combinatorial approaches. The incorporation of C7R into GD2.CARTs appears to improve clinical benefit and radiographic tumor regression in some patients, signifying the potential of advances in CART engineering to yield more effective clinical responses. Building upon these promising results in larger patient cohorts is warranted to confirm these findings and establish the role of C7R-GD2.CARTs in the treatment of GD2-expressing pediatric CNS tumors.

## Data Availability

The study protocol and clinical data collected for this study are included in the study Supplement. Additional deidentified data will be made available for approved data sharing requests that describe a methodologically sound proposal.

## APPENDIX

**TABLE A1.** Tumor Characteristics and Treatment Summary

Patient No.	Dose Level	Age, Years	Diagnosis	Tumor Molecular Profile	Methylation Class	GD2 Proportion Positive	Neurologic Deficit (CTCAE grade)	Ventricular Diversion/Access	Radiation Therapy	Pre-Enrollment Chemotherapy	Poststudy Therapy
1	0	17	DMG K27M	*H3-3A* p.K27M; *FGFR1*; *TP53*; *ATRX* mutations	DMG, K27	4	Muscle weakness (3)	Ommaya	60 Gy focal	None	None
2	0	4	ATRT	*SMARBC1* loss	Tissue not available	4	Muscle weakness (1) EOM paresis (1) Ataxia (1)	Ommaya	54 Gy focal 36 Gy CSI	SJATRT regimen B1	ALRN6924 (sulanemadlin)
3	0	13	DMG K27M	*H3-3A* p.K27M; *PTEN; TP53; ATRX* mutations	Tissue not available	Tissue not available	Ataxia (3)	Ommaya	36 Gy CSI	None	Temsirolimus/avapritinib/everolimus
4	1	14	Medulloblastoma	Non-WNT/non-SHH; *MYC*/*MYCN* not amp	Tissue not available	4	Urinary incontinence (1) Muscle weakness (1) Ataxia (3)	Ommaya	23.4 Gy CSI + primary boost 30 Gy focal 21.6 Gy CSI	ACNS0331 Cy/VP16/Thal/Cox2i/fenofibrate/Bev/IT-AraC ×6 SJDAWN Stratum A (ribociclib/gemcitabine) ×4 TMZ/Bev/irinotecan ×2	None
5/10^a^	1/2	11/12	DMG K27M	*H3-3A* p.K27M; *NF1* mutations	Not determinable	Tissue not available	EOM paresis (3) Headache (2) Steroid use (NA)	VP Shunt	54 Gy focal	None	NA
6	1	11	DMG K27M	*H3-3A* p.K27M; *PDGFRA; NF1; TERT; FH* mutations	DMG, K27	Tissue not available	Facial nerve disorder (1)	VP Shunt	54 Gy focal	None	AMXT DFMO
7	2	18	Medulloblastoma	Non-WNT/non-SHH; *MYC/MYCN* not amp	Tissue not available	4	Ataxia (1)	Ommaya	36 Gy CSI + primary boost	CCV/Cy ACNS0821	Bev/VP16/fenofibrate/Cox2i/intrathecal Ara-C and VP16
8	2	15	DMG K27M	*H3-3A* p.K27M; *PTPRZ1::MET fusion; TP53*, *KDR, KIT, MET, mutations; PDGFRA* amp	DMG, K27	3	EOM paresis (1) Facial nerve disorder (1) Dysarthria (1) Muscle weakness (2) Gait disturbance (2)	Ommaya	54 Gy focal	None	None
9	2	11	DMG K27M	H3-3A (IHC); TP53 (IHC)	Tissue not available	Tissue not available	Asymptomatic	Ommaya	54 Gy focal 30 Gy focal	Temozolomide Bevacizumab ONC201	None
11	2	10	DMG K27M	*H3-3A* p.K27M; *PIK3CA; TP53* mutations	Tissue not available	4	Dysarthria (2) Gait disturbance (1)	Ommaya	59.4 Gy focal	None	None
12	2	12	DMG K27M	*H3-3A* p.K27M; *PIK3CA*; *ACVR1* mutations	Tissue not available	0	EOM paresis (3) Facial nerve disorder (1) Muscle weakness (2) Gait disturbance (2)	Ommaya	60 Gy focal	ONC206	None

NOTE. GD2 percent positivity: 0 = no staining, 1 = 1%-25%, 2 = 26%-50%, 3 = 51%-75%, 4 = 76%-100%.

Abbreviations: Ara-C, cytarabine; Bev, bevacizumab; CCV, carboplatin, cisplatin, vincristine; Cox2i, celecoxib; CSI, craniospinal irradiation therapy; Cy, cyclophosphamide; DL, dose level; DMG, diffuse midline glioma; EOM, extra-ocular muscle; Irino, irinotecan; Thal, thalidomide; TMZ, temozolomide; VP16, etoposide.

^a^ Patients 5 and 10 represent the same individual who received two infusions at DL 1, then re-enrolled to DL 2 to receive two additional infusions to date with partial response of tumor.

**TABLE A2.** Patient Vignettes Highlighting Clinical and Radiographic Response

Patient 5/10 An 11-year-old female presented with headaches, vomiting, diplopia, and wide-based gait. MRI revealed a 3.3 × 3-cm tumor of the thalamus, extending to midbrain and tectum, with obstructive hydrocephalus. She proceeded to gross total resection and VP shunt placement, with a diagnosis reported as pilocytic astrocytoma with anaplastic transformation, harboring *H3-3A* p.K27M and *NF1* mutations (Table A1) One month after adjuvant focal radiotherapy, she presented with progressive right exotropia, bilateral ptosis, and generalized weakness. MRI revealed a new 2.3 × 2.5-cm enhancing tectal lesion. Neurologic symptoms persisted on dexamethasone 0.08 mg/kg/day 8 weeks after radiotherapy, at which time she received C7R-GD2.CARTs (1 × 10^7^ cells/m^2^). Within a week after infusion, she developed mild headache, worsening ptosis/exotropia, impaired word finding, and slurred speech (grade I TIAN), improving with 48 hours of anakinra treatment. Improvement of diplopia/ptosis, gait stability, and speech tempo was noted by 3 weeks of CART infusion. At 7 weeks, she received a second cycle of C7R-GD2.CARTs at DL1. She again developed blurry vision and perturbed speech after 1 week, resolving after 48 hours of anakinra. Preexisting neurologic deficits continued to improve, and dexamethasone was weaned off 8 weeks into the second cycle. She later re-enrolled (as patient 10) onto the expansion phase (3 × 10^7^ cells/m^2^) and has received two additional treatment cycles without severe toxicity. Serial MRIs demonstrated continued decrease in tumor size to 1.7 × 1.7 cm before the third cycle, and 0.8 × 0.8 cm before the fourth cycle (Fig 2B), meeting the definition for partial response. At the time of this report, she remains enrolled over 1 year from initial C7R-GD2.CART therapy with sustained clinical improvement.
Patient 8 A 15-year-old female presented with 2 weeks of diplopia, right CN7 palsy, slurred speech, dysphagia, right-sided numbness/weakness, and gait instability. MRI demonstrated a 4.3 × 4.5-cm pontine mass, and biopsied tissue was consistent with H3K27-altered DMG. After focal radiation therapy, the tumor decreased to 4.1 × 3.8 cm; however, neurologic symptoms persisted. Seven weeks after completion of radiation therapy, she received C7R-GD2.CART cells at DL2 (3 × 10^7^ cells/m^2^). Three days after the initial infusion, she developed fever associated with worsening neurologic symptoms (grade 1 CRS and TIAN). CRS resolved after one dose of tocilizumab and neurologic findings returned to preinfusion baseline after 7 days of anakinra. Twelve days from first cell infusion, she experienced rebound worsening of diplopia, right CN7 palsy, right hemiparesis, and gait instability, managed with 3 days of anakinra. Approximately 3 weeks from C7R-GD2.CART cell infusion, all neurologic deficits began to improve. Seven weeks after first infusion, MRI demonstrated reduction in tumor size to 2.4 × 1.4 cm. She proceeded to a second treatment cycle, developing mild dysmetria and gait instability after 10 days, but not CRS. Four weeks into the second cycle, she regained the ability to ambulate independently, and had near resolution of diplopia and slurred speech. She proceeded to a third C7R-GD2.CART cell cycle, however, exhibited clinical and radiographic progression 5 weeks from treatment.

Abbreviations: CART, chimeric antigen receptor; CRS, cytokine release syndrome; DL, dose level; DMG, diffuse midline glioma; MRI, magnetic resonance imaging; TIAN, tumor inflammation–associated neurotoxicity; VP, ventriculoperitoneal.

**TABLE A3.** Characteristics of Infused Cell Lines

Patient No.	Product	Positive C7R, %	Positive GD2, %	Positive GD2+C7R, %	Cytotoxicity
1	GD2.CAR	NA	96.7	NA	88.1
2	GD2.CAR	NA	76.4	NA	74
3	GD2.CAR	NA	34.5	NA	43
Mean GD2.CAR		NA	69.2	NA	68.4
4	C7R-GD2.CAR	61.5	77.1	49.4	66.6
5/10	C7R-GD2.CAR	65.7	76.7	52.3	44.3
6	C7R-GD2.CAR	65.4	95.5	60.6	63.2
7	C7R-GD2.CAR	80.6	80.1	66.2	44.9
8	C7R-GD2.CAR	60.2	88.0	54.3	77.4
9	C7R-GD2.CAR	61.1	81.4	50.8	64.6
11	C7R-GD2.CAR	53.5	81.0	45.1	41.3
12	C7R-GD2.CAR	29.7	76.5	23.6	65.5
Mean C7R-GD2.CAR		59.7	82.0	50.3	58.5

NOTE. Transduction efficiency as measured by percent of manufactured chimeric antigen receptor cells positive for C7R, GD2, and GD2+C7R combined and cytotoxicity as measured by chromium-release assay (20:1 effector to target ratio, minimum release criteria of 20% for GD2.CAR and C7R).
